# Comprehensive insights into pathogenesis, diagnosis, treatment, and prognosis in adult autoimmune enteropathy

**DOI:** 10.1186/s13023-025-03731-2

**Published:** 2025-05-02

**Authors:** Muhan Li, Tianming Xu, Gechong Ruan, Chengzhu Ou, Bei Tan, Shengyu Zhang, Xiaoqing Li, Yan You, Weixun Zhou, Ji Li, Jingnan Li

**Affiliations:** 1 Department of Gastroenterology, Peking Union Medical College Hospital, Chinese Academy of Medical Sciences and Peking Union Medical College, Beijing, China; 2 Department of Pathology, Peking Union Medical College Hospital, Chinese Academy of Medical Sciences and Peking Union Medical College, Beijing, 100730 China

**Keywords:** Autoimmune enteropathy, Clinical presentation, Pathological features, Treatment

## Abstract

Autoimmune enteropathy (AIE) constitutes a diverse array of disorders characterized by immune dysregulation and gastrointestinal manifestations, chiefly chronic diarrhea. Due to the small number of reported cohorts, the current knowledge and understanding of adult-onset AIE is rare compared with pediatric or syndromic AIE. Pathogenesis might involve genetic predisposition, aberrant immune homeostasis, comorbidities of autoimmune diseases and environmental trigger. Diagnosis relies on a comprehensive assessment encompassing clinical evaluation, laboratory tests, endoscopic findings and histopathological features, yet distinguishing AIE from other disorders with similar presentations poses diagnostic challenges. Treatment strategies predominantly center on immunosuppressive therapies, exhibiting varied efficacy among individuals. Supportive treatment and prevention and management of complications are also important for prognosis. The prospectives of future researches need to explore the genetic and immunological mechanism, the diagnostic modalities and the treatment strategies to improve patient outcomes.

**Supplementary Information:**

The online version contains supplementary material available at 10.1186/s13023-025-03731-2.

## Introduction

From a broad perspective, autoimmune enteropathy (AIE) may actually encompass a large variety of disorders characterized by immune dysregulation with gastrointestinal involvement and presenting as chronic diarrhea. Some researchers have proposed that the term of autoimmune enteropathy should be rectified as autoimmune enteropathies because of its heterogeneous manifestations, which can be further subdivided into at least five categories: pediatric primary AIE; pediatric syndromic AIE; adult primary/sporadic AIE; adult secondary/iatrogenic AIE; paraneoplastic AIE [1]. The comprehension of adult AIE was less known compared with pediatric AIE or syndromic AIE [2, 3]. In children presenting with prolonged and refractory diarrhea, AIE stands out as one of the most common diagnosis, though with an estimated incidence of less than 1 in 100,000 [1]. And the two rare but well-studied syndromic AIE are immune-dysregulation poly-endocrinopathy enteropathy X-linked (IPEX) syndrome occurring in 1 in 1,600,000, and autoimmune poly-endocrinopathy, candidiasis, and ectodermal dystrophy (APECED) syndromes that is also named as autoimmune polyglandular syndrome type 1 (APS-1) occurring in 1 in 80,000–130,000 [1, 4]. Up to this point, the literature has documented about 200 cases of adult primary/sporadic AIE. Yet this may not fully depict the actual prevalence accurately because medical professionals’ unfamiliarity with adult AIE could lead to clinical cases being overlooked or misdiagnosed as other disorders causing villous atrophy. Therefore, this review will pivot towards the relatively underexplored adult primary AIE, and elaborate on its pathogenesis, clinicopathologic manifestations, diagnosis and differential diagnosis, treatment and prognosis, hoping to deepen the level of understanding of this rare disease.

## Pathogenesis

The exact etiology of adult AIE remains unclear, but as far as we know, the pathogenesis of adult AIE seems to reflect a spectrum of generalized immune dysregulation [5], with genetic [6] and environmental [7] factors might contribute to some extent.

### Genetic predisposition

Genetic variants appear to be involved in pathogenesis of AIE, not only in children but also in adult. But Unlike IPEX syndrome and APECED syndrome, which have well-established pathogenic genes and elucidated the mechanisms of abnormal immune reactions resulting from these gene mutations, adult AIE cannot be fully explained by a single or a couple of gene mutation or be solely attributed to genetic factors. There appears to be significant involvement of acquired factors in addition to genetic predisposition. Latest genetic research shows that pathogenic variants can be identified in 20/48 (41.6%) AIE patients, including CTLA-4, TNFRSF13B, STAT3, LRBA, CIS, STAT1, NFBK1, ICOS [6]. Adult AIE patients harboring pathogenic gene variants experienced earlier onset of enteropathy, with more rapidly progressing malignant gastrointestinal disorders [6]. These genes all play an important role in normal immune response, and detailed description of each of these genes are shown in Table 1. Whole exome sequencing found defects in CTLA4, STAT1, STAT3 harm regulatory T cell function and could present as IPEX-like phenotype [1]. Interestingly, in CHAI (CTLA-4 haploinsufficiency with autoimmune infiltration), enteropathy is more frequent in patients with a missense mutation (84.6%) compared to those with nonsense or insertion/deletion frameshift mutations (15.6%, *p* = 0.028) [8]. Defects in other genes, such as TNFRSF13B, LRBA, NFKB1, ICOS, are closely related to immunodeficiency. Given that the proportion of adult AIE patients with immunoglobin deficiency in this study cohort reached 67%, the genetic results are consistent with this clinical information. It should be noted that AIE patients with immunodeficiency are often difficult to be distinguished from common variable immunodeficiency (CVID), which is a difficult problem encountered in clinical work and scientific research. Meanwhile, it is still unknown whether adult AIE with immunocompetency has these above-mentioned genetic mutations.

**Table 1:** Gene variants associated with autoimmune enteropathy

Condition	Associated genetic variants	Gene Function	Clinical presentation related to the pathogenic genetic variants
IPEX	*FOXP3*	Regulatory T cells function	X-linked, endocrinopathy (type 1 DM, thyroiditis), AIE, dermatitis (eczema)
APECED	*AIRE*	Immune tolerance and elimination of autoreactive T cell	Autosomal recessive, candidiasis, hypoparathyroidism, adrenal insufficiency
Adult AIE	*CTLA4*	Regulatory T cell function, transmits inhibitory signal to T cells	Celiac disease, insulin-dependent DM, Hashimoto thyroiditis, systemic lupus erythematosus, CHAI
	*TNFRSF13B*	Play a crucial role in humoral immunity by interacting with a TNF ligand	Immunoglobulin A deficiency 2, CVID 2
	*STAT3*	transcription activators	Infantile-onset multisystem autoimmune disease (insulin-dependent DM, AIE, or celiac disease, autoimmune hematologic disorders), hyper-immunoglobulin E syndrome.
	*LRBA*	LRBA regulates CTLA4 expression, LRBA deficiency leads to reduced CTLA4 in regulatory T cells and activated T cells.	CVID 8, LATAIE
	*C1S*	A major constituent of the human complement subcomponent C1	C1s deficiency, Ehlers-Danlos syndrome periodontal type 2
	*STAT1*	Helper T cell response	Immunodeficiency
	*NFKB1*	Pleiotropic transcription factor	CVID 12
	*ICOS*	Enhances all basic T-cell responses to a foreign antigen	CVID 1

IPEX, immune-dysregulation poly-endocrinopathy enteropathy X-linked syndrome; APECED, autoimmune poly-endocrinopathy, candidiasis, and ectodermal dystrophy; FOXP3, Forkhead Box P3; AIRE, autoimmune regulator; CTLA-4, cytotoxic T-lymphocyte associated protein 4; TNFRSF13B, tumor necrosis factor receptor superfamily member 13B; STAT3, signal transducer and activator of transcription 3; LRBA, lipopolysaccharide-responsive and beige-like anchor protein; C1S, Complement C1s; STAT1, signal transducer and activator of transcription 1; NFKB1, nuclear factor kappa B subunit 1; ICOS, Inducible T Cell Co-stimulator; DM, diabetes mellitus; AIE, autoimmune enteropathy; CHAI, CTLA-4 haploinsufficiency with autoimmune infiltration; CVID, common variable immunodeficiency; LATAIE, LRBA deficiency with autoantibodies, regulatory T cell defects, autoimmune infiltration and enteropathy

### Aberrant immune homeostasis

The defective immune tolerance mechanisms and loss of immune homeostasis could potentially have a significant impact and may contribute to the development of AIE. Pediatric AIE is closely related to abnormal regulatory T-cell function caused by mutations in FOXP3, a transcription factor that regulates the development of regulatory T lymphocyte (Table 1) [9]. Through phenotypic and functional analysis of T lymphocytes of an adult primary AIE patient (26 years old), regulatory T lymphocytes with quantitative deficit of FOXP3 expression are linked to uncontrolled IL-17 production by intraepithelial lymphocyte (the majority being CD8 + T cells), which is augmented by transforming growth factor-β (TGF-β) [10]. The detection of other genetic mutations associated with regulatory T cell function in adult AIE patients further highlights the important role of regulatory T cells in pathogenesis (see “Genetic variants” part). Besides, aberrant expression of certain human leukocyte antigen (HLA) class II molecules and abnormal expression of self-antigens on epithelial cells further impair immune tolerance and trigger T cells, leading to immunological destruction of enterocytes through apoptosis or other cytotoxic effects in AIE [4]. Calcineurin inhibitors, most commonly represented by cyclosporine and tacrolimus, function by inhibiting T-cell activation factors such as IL-2. And biological agents such as infliximab specifically target TNF-α so as to induce apoptosis of activated T cells and macrophages. Their efficacy reported in adult AIE and even in adult refractory AIE further supports the role of abnormal immune cells activation in disease progression (see Supplementary Tables 1 and “Treatment and Prognosis” part) [11–13].

### Comorbidities of autoimmune diseases and increased titer of autoantibodies

The theory that AIE is actually an immune disorder is further backboned by the association between AIE and thymoma [14–17], cancer in a pivotal immune-regulatory organ, together with the association between AIE and many autoimmune diseases [18], such as hypothyroidism, primary sclerosing cholangitis, autoimmune hemolytic anemia, autoimmune hepatitis, autoimmune gastritis, rheumatoid arthritis, type 1 diabetes, and so on. In addition, AIE is also in association or combined with immunodeficiency such as CVID [5]. In a cohort with 48 cases, primary immunoglobulin deficiency and lymphopenia (particularly B lymphopenia) were present in 67% and 70% of patients, respectively [6]. However, there are also many adult AIE patients who mainly present with gastrointestinal symptoms without other immune deficiencies or endocrine involvement. These individuals might need to be further considered as a separate disease entity, with far more distinct features from syndromic AIE (see Table 3 and “Clinical manifestations” part).

It is well known that AIE is closely related to anti-enterocyte (AE) and anti-goblet cell (AG) antibodies, but the pathogenic contribution of AE and AG to the development of AIE remains uncertain, with some suggesting these antibodies may merely be secondary byproducts of mucosal injury, which is supported by the fact that AE and AG can also be found in other enteropathy (AE are present in 7.7% celiac disease, 20% enteropathy-associated T-cell lymphoma [19], AG in up to 28% celiac disease [20, 21]). AIE-related 75-kilodalton protein (AIE-75) has been identified as an antigen for autoantibody in AIE patients [22–24]. AIE-75 mainly distributed in the epithelial cells of the luminal surface and the upper half of the crypts of the intestine [22], takes part in tight-junction integrity and cytoskeleton formation [24, 25]. Therefore, antibodies to AIE-75 would impair intestinal permeability and may finally contribute to secretory diarrhea [15, 16]. However, these antibodies are not all essential for AIE diagnosis according to variable diagnostic criteria(see “Diagnosis and differential diagnosis” part).

### Environmental trigger

It is reasonable to speculate that adult primary AIE is more influenced by acquired factors than pediatric and syndromic AIE. Surgery such as proctocolectomy in ulcerative colitis (UC) patients may serve as a trigger of a dysfunctional immune response to microbial shift, which results in clinical and pathological features similar to AIE [7, 11, 26]. Post-colectomy enteritis is a rare but serious development after surgery for UC, sometimes with clinical presentation of high ileostomy volumes or severe diarrhea and pathological presentation of apoptotic bodies and decreased goblet cells [7]. Hence propose the possibility that post-colectomy enteritis may actually a kind of immune-dysregulation disorder thus presents with AIE-like features. Perhaps they all reflect a broad immune dysregulation leading to gastrointestinal involvement, and can be regarded as a more general form of autoimmune enteropathy.

## Clinical manifestations

Two independent investigators (Muhan Li and Tianming Xu) searched PubMed and Wanfang databases using the keyword “Autoimmune enteropathy” to identify all AIE cases reported in the literature published before April 2024. All 208 adult AIE cases were finally reviewed, and their data including basic demographic characteristics, the diarrhea characteristics, other clinical features, endoscopic characteristics, histopathologic features, effect of treatment and follow-up information were collected, as shown in Table 2. Because the information recorded in the literature is not sufficiently detailed, the clinical features of some cases were missing. Therefore, cases with detailed and definitive descriptions of clinical features are termed as effective cases. The median age of diagnosis was 49.0 years (IQR: 36.5 ~ 60.5 years, IQR stands for interquartile range) and there was slightly female predominance in adult AIE patients (female: male = 1.3:1) (Table 2, Supplementary Table 1). The median duration of diarrhea was 6.0 (IQR: 2.9 ~ 24.0) months, with a median weight loss of 16.0 (IQR: 10.0 ~ 25.8) kg and mostly associated with a significantly low body mass index (BMI). Frequent diarrhea profoundly impacts patients’ normal lives, as cases with bowel movements equal to or exceeding 10 times per day constitute 83% of the total. The stool volume per day of all patients can reach 1,000 ml, with severe cases reaching up to 10,000 ml. Digestive symptoms may be severe to the extent that rely on parenteral nutrition in some cases.

**Table 2:** Characteristics of reported AIE in literature

Clinical features	All cases*	Results
Age (years) at Dx	129	49.0 (IQR: 36.5~60.5)
Gender	145	Female/Male = 82/63 = 1.3/1
Comorbidities	178	CVID/hypogammaglobulinemia/agammaglobulinemia in 48 cases (27%)
Diarrhea duration (months)	78	6.0 (IQR: 2.9 ~ 24.0)
Weight loss (kg)	48	16.0 (IQR: 10.0 ~ 25.8)
Stool frequency (/day)	40	≥ 10+/day accounts for 83% (33/40)
Stool volume (/day)	27	1000 ~ 10,000 ml
Character of stool	77	Mostly watery, without blood or mucus, sometimes steatorrhea or protein-losing.
D-xylose test	20	(+) in 19 cases (95%)
Fasting test	23	(–) in 19 cases (83%)
Gluten-free diet	95	Ineffectiveness in 82 cases (86%)
HLA-DQ2/8	90	(+) in 45 cases (50%)
AE antibody	96	(+) in 50 cases (52%)
AG antibody	68	(+) in 9 cases (13%)
Celiac antibody profiles	136	(–) in 111 cases (82%), tTG + in 11 cases (8%), AGA + in 9 cases (7%), tTG and AGA + in 4 cases (3%), DGP + in 1 case (1%)
Villous atrophy	170	Reported in 150 cases (88%)
IEL	149	IEL in 76 cases (51%), minimal IEL in 73 cases (49%)
Increased apoptotic bodies	122	Reported in 60 cases (49%)
Decreased or absent goblet cells	107	Reported in 55 cases (51%)
Decreased or absent Paneth cells	107	Reported in 35 cases (33%)
Neutrophil infiltration	107	Reported in 60 cases (47%)
Corticosteroids therapy	167	Used in 148 cases (89%)
Immunosuppressant therapy	155	Used in 63 cases (41%)
Biological agent therapy	160	Used in 51 cases (32%)
Follow-up outcomes	164	Death in 23 cases (14%)

Dx, diagnosis; HLA, human leukocyte antigen; AE, anti-enterocyte antibody; AG, anti-goblet cell antibody; IEL, intraepithelial lymphocytosis, means > 40 lymphocytes per 100 epithelial cells; Minimal IEL, means less than 40 lymphocytes per 100 epithelial cells; AGA, anti-gliadin antibodies; tTG, tissue transglutaminase, DGP, deamidated gliadin protein

* The number of effective cases reported in literature until 2024. Because the information recorded in the literature is not sufficiently detailed, the clinical features of some cases were missing. Therefore, cases with detailed and definitive descriptions of clinical features are termed as effective cases

The character of their stool is mostly profuse and watery, without blood or mucus, but sometimes steatorrhea or protein-losing diarrhea. Although there are few reports documenting the results of D-xylose tests, current findings suggest significant impairment of intestinal absorption function in AIE patients (95%, 19/20). The information of fasting test was also incomplete in many literatures, though available data showed similar conclusion as our reported Chinese AIE cohort [27]: AIE is mainly secretory diarrhea, which is backboned by the fact that the fasting test is inefficient in the vast majority of cases (83%). Gluten-free diet is also generally ineffective in these cases (86%, 82/95), which is contrast to celiac disease (Table 2). The percentage of HLA-DQ2 or DQ8 is 50%, but celiac antibody profiles is negative in 82% cases. What’s more, the percentage of AE antibody positivity (52%) is higher than AG (13%).

Due to the limited understanding of AIE, many questions remain contentious, such as whether adult patients with concomitant immunodeficiency should be diagnosed as AIE, which has not yet reached a consensus. Many patients reported in the literature have concomitant immunodeficiency (48 cases in total), including CVID, hypogammaglobulinemia, agammaglobulinemia. Therefor we have separately summarized the clinical characteristics of adult patients with and without immunodeficiency. Due to incomplete information in many literature reports, only age at diagnosis, gender, AE antibodies, and AG antibodies had relatively sufficient data for statistical analysis (Table 3). According to the results of Wilcoxon rank sum test or chi-square test, the age at diagnosis of adult AIE patients with concomitant immunodeficiency was significantly younger than those without concomitant immunodeficiency (*p* = 0.030). There was also a discrepancy in gender distribution between the two groups (*p* = 0.056, marginally significant), with a higher proportion of male in immunodeficient group and a higher proportion of females in the other. Interestingly, the positivity rate of AE antibodies in adult AIE patients with concomitant immunodeficiency was significantly lower than those without (*p* < 0.001), whereas AG antibodies did not exhibit this trend (Table 3). So further studies are needed to clarify the difference in the clinical manifestations between adult AIE patients with and without primary immunodeficiency.

**Table 3:** Characteristics of reported AIE in literature with or without immunodeficiency

Clinical features	All cases with immunodeficiency*	Results of cases with immunodeficiency	All cases without immunodeficiency*	Results of cases without immunodeficiency	*P* value
Age (years) at Dx	16	41.5 (IQR: 28~54.8) ^$^	113	50.0 (IQR: 38~62) ^$^	0.030^$^
Gender	16	Female/Male = 6/10 = 0.6/1	102	Female/Male = 64/38 = 1.68/1	0.056^#^
AE antibody	14	(+) in 1 case (7%)	56	(+) in 35 cases (63%)	< 0.001^#^
AG antibody	13	(+) in 1 case (8%)	29	(+) in 7 cases (24%)	0.398^#^

Dx, diagnosis; HLA, human leukocyte antigen; AE, anti-enterocyte antibody; AG, anti-goblet cell antibody; IEL, intraepithelial lymphocytosis, means > 40 lymphocytes per 100 epithelial cells; Minimal IEL, means less than 40 lymphocytes per 100 epithelial cells; AGA, anti-gliadin antibodies; tTG, tissue transglutaminase

* The number of effective cases reported in literature until 2024. Because the information recorded in the literature is not sufficiently detailed, the clinical features of some cases were missing. Therefore, cases with detailed and definitive descriptions of clinical features are termed as effective cases

$ Continuous variables that does not fit the normal distribution are shown as the median (IQR: 25th percentile point ~ 75th percentile point) with IQR means interquartile range. Wilcoxon rank sum test was used for the difference between the two groups of measurement data not conforming to normal distribution. A *p* < 0.05 was considered to indicate statistical significance in the SPSS 26.0 analysis

# Categorical variables were expressed as the percentage. The chi-square test is employed for comparing rates between two groups. When the expected frequencies are all greater than 5, the *p*-value is derived from the Pearson chi-square test. In cases where there are expected frequencies less than 5, the *p*-value is obtained from Fisher’s exact test. A *p* < 0.05 was considered to indicate statistical significance in the SPSS 26.0 analysis

## Endoscopic findings and pathologic features

Endoscopic changes are unconspicuous and nonspecific, which cannot accurately serve as evidence for distinguished diagnosis from other villus blunting disease alone. But for experienced endoscopists, careful observation of the duodenum during gastroscopy and the terminal ileum during colonoscopy is really suggestive and could remind them the susceptibility of AIE. Actually, the main manifestations of adult AIE patients in the duodenum include edema, villous blunting, and mucosal hyperemia, with some other features (nodular changes, erosion, “scalloped” folds, mosaic pattern, and occasionally ulcers) can also be observed [27].Similar presentations are noted in the terminal ileum as in the duodenum. Involvement of other segments of the digestive tract was observable, including stomach and colon [16, 17, 27–30]. In cases where endoscopic findings lack obvious abnormalities or typical features of other diseases (such as Crohn’s disease, tuberculosis, etc.), consideration of the possibility of AIE is warranted, and the next step is to take biopsies. Biopsy from the duodenum during gastroscopy and terminal ileum during colonoscopy is decisive for final diagnosis and differential diagnosis. Capsule endoscopy is also suggestive for diagnosis, but it is important to carefully interpret capsule endoscopy results to avoid missing lesions that are not prominent [31]. However, capsule endoscopy cannot be used for biopsy, which is not advantageous for the diagnosis of AIE. In addition, the significance of endoscopic examination during follow-up of adult AIE patients is still unclear and there is a lack of standardized follow-up protocol.

The small intestinal mucosa of AIE patients showed characteristic pathological changes, including villous blunting, mononuclear inflammatory cell infiltration, increased apoptotic bodies, minimal intraepithelial lymphocytosis and reduced goblet/Paneth cells. Among all these cases reported in the literature, villous atrophy is the most prevailing feature (88%, Table 2), manifesting as partial or complete blunting both in duodenal and ileal biopsies. Various degrees of lymphocyte and plasma cell infiltration were commonly noted, with infiltration typically more pronounced in the lamina propria than in the deep crypts. The intraepithelial lymphocytosis (IEL) means more than 40 lymphocytes per 100 epithelial cells, whereas minimal IEL represents less than 40 lymphocytes per 100 epithelial cells. IEL were reported in 51% AIE patients in 2018 cohort. Apoptotic bodies are elevated in around half of cases documented in the literature (49%) (Table 2). 55 cases have been reported with decreased or absent goblet cells, and 35 cases reported with decreased or absent Paneth cells. Neutrophil infiltration is also found in 47% AIE patients, and even more prevailing (69% in ileum, 100% in duodenum) in our previous reported cohort [27]. More detailed information including clinical features, endoscopic characteristics, histopathologic features, effect of treatment and follow-up outcome can be found in Supplementary Table 1.

## Diagnosis and differential diagnosis

The clinical and histopathological manifestations can vary, and some individuals may present with other misleading comorbidities, making diagnosis and differential diagnosis challenging. As reported, median duration of symptoms before making AIE diagnosis was 1.5 years [5]. So far, there are 4 versions of criteria in adult AIE patients have been proposed (Table 4 [27]). The criteria in 1985 primarily focused on describing clinical characteristics of AIE. The 2007 criteria, which are the most widely used, concurrently consider clinical features, pathological characteristics, differential diagnosis, and antibody results. The 2018 standards are more comprehensive, further refining diagnostically valuable pathological features, especially absence of goblet or Paneth cells, and detailing specific items for differential diagnosis. The 2018 standards still do not emphasize the value of AE or AG antibodies. Later, the expert consensus in 2022 places greater emphasis on patients’ clinical and serological characteristics, designating AE antibodies as a necessary diagnostic criterion, while pathological features only serve as supportive criterion, and consensus regarding the diagnostic value of many pathological characteristics has not been reached. However, as mentioned above, AE and AG antibodies were not specific and sensitive enough as the necessary criterion. For example, our previous study showed many adult AIE patients with negative AE and AG antibodies, and would be excluded according to the 2022 consensus [27].

**Table 4:** Different criteria proposed for diagnosing autoimmune enteropathy

Unsworth & Walker-Smith proposed in 1985	S. Akram et al., proposed in 2007	A. Sharma et al., proposed in 2018	A. Schiepatti et al., proposed in 2022
1. protracted diarrhea and severe enteropathy; 2. no response to exclusion diet or total parenteral nutrition; 3. evidence of predisposition to autoimmune disease (presence of circulating autoantibodies and/ or associated disease also thought to be autoimmune); 4. no severe immunodeficiency.	1. Adult-onset chronic diarrhea (> 6 weeks’ duration) 2. Malabsorption 3. Specific small bowel histology Partial/complete villous blunting Deep crypt lymphocytosis Increased crypt apoptotic bodies Minimal intraepithelial lymphocytosis (IEL means > 40 per 100 epithelial cells) 4. Exclusion of other causes of villous atrophy including celiac disease, refractory sprue, and intestinal lymphoma 5. anti-enterocyte (AE) and/or anti–goblet cell (AG) antibodies Criteria 1 ~ 4 are required for a definite diagnosis of AIE. Presence of AE and/or AG antibodies is an important diagnostic support, but their absence does not exclude the diagnosis of AIE.	1. adult-onset protracted diarrhea (> 6 weeks) not responsive to any dietary exclusion; 2. associated with specific small- bowel histology villous atrophy minimal IEL increased crypt apoptotic bodies absence of goblet or Paneth cells 3. systematic exclusion of other causes of villous atrophy including CD or RCD (based on combination of celiac serology, HLA typing, histopathology features, and response to GFD), drug-induced enteropathy (based on history of starting medication and its correlation with symptoms, duration of treatment, histopathology and effect after discontinuation), common variable immunodeficiency-associated enteropathy (based on total immunoglobulin titers and histopathology [presence of plasma cells, lymphoid aggregates]), collagenous sprue and colitis (based on histopathology), and intestinal lymphoma.	the following criteria must be satisfied for the diagnosis: 1.Severe malabsorption symptoms (chronic diarrhoea, weight loss, nutritional deficiencies and electrolyte imbalance) unresponsive to any dietary restriction. 2.Frank villous atrophy unresponsive to any dietary restriction. 3.IgA/IgG positive enterocyte antibodies (indirect immunofluorescence on human/monkey jejunum). 4.Negative coeliac serology. 5.Exclusion of other causes of villous atrophy. The following criteria were considered supportive for the diagnosis: 1.History of associated autoimmune conditions. 2.Clinical response to immunosuppressive treatments. 3.Deep crypt lymphocytosis and/or plasma cells infiltration, neutrophilic cryptitis ± crypt microabscesses and lack/decrease of Paneth cells on duodenal histology. 4.Positive serum anti-AIE 75KD antibodies (ELISA) or nonorgan specific autoantibodies. No consensus was found for the following items: absence of severe immunodeficiencies, diagnostic role of serum antigoblet cells antibodies, involvement of other sites of the GI tract and some duodenal histopathological features (include intraepithelial lymphocytes count, crypt hyperplasia and crypt apoptotic bodies, lack of gamma-delta T cells and depletion of goblet cells)

IEL: Intraepithelial lymphocyte; CD: celiac disease; RCD: refractory celiac disease; HLA: human leukocyte antigen; GFD: gluten-free diet; ELISA: enzyme-linked immuno sorbent assay

In general, we prefer the 2007 or 2018 criteria, where diagnosis typically involves a combination of clinical evaluation, laboratory tests and most importantly histopathological examination of intestinal biopsies. The inclusion of endoscopic findings in diagnostic criteria remains to be determined. Whether patients with concomitant immunodeficiency should be included in the diagnosis of AIE yields different answers depending on these constantly evolving criteria. CVID was listed as the comorbidity of AIE in some studies. However, we might reconsider CVID associated enteropathy as an independent condition different from AIE. CVID patients usually have past medical history of recurrent respiratory or gastrointestinal infection, low titer of IgG, and decrease or absence of plasma cells in intestinal biopsies [32], which might help the discrimination between AIE and CVID. The analysis results from Table 3 indicate that the two groups of adult AIE patients, with and without immunodeficiency, exhibit distinct characteristics, further supporting the differentiation of these two populations in future diagnosis and studies. Besides, due to the heterogeneity of AIE diseases, it may be necessary to refine both narrow and broad criteria in the future, with the difference lying in the extent of defining the disease scope.

Beyond CVID, the differential diagnosis between AIE and other diseases manifested as villous atrophy, such as Olmesartan-Induced Enteropathy, celiac disease, Whipple’s disease, lymphoma [33], remains challenging, which leads to many mis-diagnosis and missed diagnosis. Olmesartan can induce all kinds of AIE-like manifestations, including the absence of goblet cells [34, 35], which makes the differential diagnosis more confusing and mainly depends on the patient’s medication history. As for distinguishing celiac disease, careful counting of the number of intraepithelial lymphocytes is a powerful tool [18]. In contrast to celiac disease where IEL is present in 100% of cases, IEL is observed in only 1/3 ~ 1/2 of AIE patients [18]. Moreover, we found that four main histological patterns are all accompanied by the reduction or absence of goblet cells and Paneth cells, including celiac-like and GvHD-like patterns (GvHD means graft-versus-host disease) [27]. Therefore, we propose that the reduction in goblet cells and Paneth cells could serve as a crucial factor in distinguishing celiac disease, particularly refractory celiac disease [27].

In general, the diagnosis of AIE should be suspected in an adult with chronic secretory diarrhea, refractory to any exclusion diet, with negative celiac serology, negative HLA-DQ2/8, positive autoantibodies, comorbid autoimmune disease, no olmesartan medication history, and no overseas travel, particularly after exclusion of CVID [1].

## Treatment and prognosis

Immunosuppressive medications, including corticosteroids, immunomodulators and biologic agents have been used to achieve remission in AIE (Table 2). Our previous study found the majority of patients achieved clinical response or remission from diarrhea in a median of 5 days (IQR: 3 ~ 20) after initiating glucocorticoid therapy (14/16, 88%) [27]. The favorable efficacy of budesonide has also been observed in other centers (85% patients reached clinical response) [18].Tacrolimus is the most commonly used immunosuppressant at our center [27]. Anti-TNF-α agents have also shown good efficacy in some AIE cases, such as infliximab [5, 13, 36] and adalimumab [37, 38]. In addition, targeted therapy based on molecular diagnosis has already achieved in adult AIE patients [6]. Research has shown that based on genetic testing results, guiding the selection of treatment medications for adult AIE patients has achieved satisfactory outcomes, including abatacept for patients carried CTLA-4 variant and JAK inhibitor ruxolitinib for patient carried STAT-3 variant [6]. This may suggest that adult AIE patients with and without genetic mutations exhibit different clinical characteristics, warranting distinct clinical pathway, treatment suggestion and follow-up procedure for each subgroup.

Treatment responses can vary among individuals, and some patients may be refractory to many kinds of immunosuppressive therapies. Some studies have reported other unusual or unconventional treatment options for AIE, including thymectomy [14], intravenous immunoglobulins [14], glutamine-supplemented parenteral nutrition and probiotics [39], autologous stem cell transplantation [19], autologous bone marrow–derived mesenchymal stromal cells [40] and isolated intestine transplantation [41]. However, these unconventional treatment methods remain limited to a minority of cases, indicating the need for further exploration.

Various complications could appear in the course of AIE disease, including hypovolemic shock, Wernicke encephalopathy, acute kidney injury, hypoalbuminemia and anemia [27]. Therefore, adequate enteral nutrition support, parenteral nutrition support, or their combination, supplementation of albumin and multiple vitamins, keeping the balance of water and electrolyte, improving anemia, and monitoring fluid intake and output and creatinine levels are all influential and crucial for the recovery of patients. It is also important to note that monitoring for adverse reactions to immunosuppressive agents should be conducted during the treatment process, so as to improve outcomes and minimize patients’ suffering.

Until now, limitations in the management of adult AIE include the absence of a standardized treatment paradigm, challenges with steroid refractory and dependent, the choice of strategy for maintenance therapy, and the high frequency of diarrhea relapse. Diarrhea relapse is a tricky but very common condition in adult AIE patients. Our previous study found the relapse-free survival rates at 6 months, 12 months, and 48 months were 62.5%, 55.6%, and 37.0%, respectively [27]. When the patient’s diarrhea relapses, a combination of immunosuppressants or switching to another kind of immunosuppressant is usually considered. Furthermore, there is a notable concern regarding the potential development of gastrointestinal malignancies [6] and lymphoma [27, 42] in AIE patients. Like celiac disease, AIE also shows increased risk of malignancy, such as enteropathy-associated T-cell lymphoma, which may be related to genetic variants [6]. The prognosis of adult AIE varies depending on the duration and severity of diarrhea, response to treatment, and the presence of complications. Overall, 23 patients died because of various causes, such as cerebral cancer metastasis [42], myocardial infarction [3], candidiasis and sepsis [43], and multiorgan dysfunction syndrome [44]. These limitations underscore the complexity and variability of AIE presentation and emphasize the need for further research to establish more effective therapeutic strategies and improve patient outcomes.

## Conclusion

Autoimmune enteropathy (AIE) is a heterogeneous group of disorders characterized by immune dysregulation and gastrointestinal involvement, primarily manifesting as chronic diarrhea. While pediatric and syndromic AIE are relatively well-understood, adult AIE remains less explored. Pathogenesis involves a complex interplay of factors including genetic predisposition, aberrant immune homeostasis, comorbidities of autoimmune diseases and environmental trigger. Typical clinical manifestation of AIE includes protracted profuse watery diarrhea together with negative fasting test, severe malabsorption, weight loss, and no response to any exclusion diet, e.g. gluten-free diet. AIE Patients combined with immunodeficiency might exhibit unique characteristics, suggesting potential complex pathophysiological interactions between primary immunodeficiency and AIE. Diagnosis relies on comprehensive consideration of clinical evaluation, laboratory tests, endoscopic findings, and histopathological examination. Differential diagnosis from other villous atrophy-related disorders presents challenges. Treatment includes immunosuppressive therapies, with varied responses among individuals, together with nutrient supports and prevention and management of complications. Diarrhea relapse and malignancy risk contribute to diverse prognoses. Limitations exist in therapeutic approaches, emphasizing the need for further research to enhance understanding and improve patient outcomes.

## Electronic supplementary material

Below is the link to the electronic supplementary material.

Supplementary Material 1

## Data Availability

All data generated or analyzed during this study are included in this published article and its supplementary information files.

## Abbreviations

AIE: Autoimmune enteropathy
IPEX: Immune-dysregulation poly-endocrinopathy enteropathy X-linked syndrome
APECED: Autoimmune poly-endocrinopathy, candidiasis, and ectodermal dystrophy
APS-1: Autoimmune polyglandular syndrome type 1
CHAI: CTLA-4 haploinsufficiency with autoimmune infiltration
CVID: Common variable immunodeficiency
TGF-β: Transforming growth factor-β
HLA: Human leukocyte antigen
AE: Anti-enterocyte antibodies
AG: Anti-goblet cell antibodies
UC: Ulcerative colitis
IQR: Interquartile range
IEL: Intraepithelial lymphocytosis
GvHD: Graft-versus-host disease
